# Reversible Wernicke encephalopathy caused by hyperemesis gravidarum in the second trimester of pregnancy: a case report

**DOI:** 10.11604/pamj.2021.40.240.30245

**Published:** 2021-12-20

**Authors:** Nadia Ben Jdidia, Sawssan Ben Halima, Hana Hakim, Sahbi Kebaili, Ines Koubaa, Hedi Chelly, Kais Chaabane

**Affiliations:** 1 Department of Gynecology and Obstetrics, University of Medicine of Sfax, Hedi Chaker Hospital, 3029, Sfax, Tunisia,; 2 Department of Radiology, University of Medicine of Sfax, Hedi Chaker Hospital, 3029, Sfax, Tunisia,; 3 Department of Intensive Care, University of Medicine of Sfax, Habib Bourguiba Hospital, 3029, Sfax, Tunisia

**Keywords:** Wernicke encephalopathy, hyperemesis, gravidarum, case report

## Abstract

Wernicke encephalopathy is a potentially life-threatening neurologic syndrome caused by acute thiamine (vitamin B1) deficiency. It is usually associated with excessive alcohol consumption. Less frequently, this syndrome can be caused by persistent vomiting. This is a case report of a 33-year-old woman diagnosed with Wernicke encephalopathy (WE) during the second trimester of pregnancy. The presence of neurological and ophthalmological symptoms in the context of hyperemesis gravidarum led us to evoke the diagnosis of WE, and it was confirmed when specific lesions were found in the brain magnetic resonance imaging (MRI). Luckily for our patient, WE was diagnosed promptly and the signs were reversible after thiamine supplementation. In conclusion, any first line care taker or midwife must know the symptoms of Wernicke encephalopathy because prompt diagnosis and treatment can lead to recovery

## Introduction

Wernicke encephalopathy (WE) is a rare, devastating and acute neurological disorder caused by thiamine (Vitamin B1) deficiency. This condition is usually a result of excessive alcohol intakes, but several cases have been found in the context of hyperemesis gravidarum which is a state of severe nausea and vomiting during pregnancy that leads to dehydration, electrolyte disorders, malnutrition and weight loss. WE secondary to hyperemesis gravidarum is uncommon. In fact, the reported prevalence of Wernicke encephalopathy is 1.2% [1]. The classical clinical triad of signs of WE includes ocular signs, cerebellar dysfunction and confusion. We are presenting a case of a pregnant woman suffering from hyperemesis gravidarium that led to WE along with spontaneous nystagmus and typical imaging findings on the brain MRI.

## Patient and observation

**Patient information:** this was a 33-year-old G2P1 woman who presented to our emergency unit at 15 weeks of gestation for symptoms of weakness, amnesia, confusion and paresthesia. She had no prior medical or surgical history, and the first pregnancy was uneventful. Moreover, our patient had no history of alcohol consumption.

**Clinical findings:** upon examination, the patient was conscious, disoriented and confused with Glasgow coma score of 13/15. The neurological examination revealed loss of equilibrium with incoordination of gait and ataxia. Blood pressure was 90/60 mmhg, pulse rate was 100/min and the patient was afebrile. There was no restriction of ocular movements, but vertical nystagmus was found. Visual acuity was preserved.

**Timeline of current episode:** the patient reported that 25 days before this visit, she had presented excessive nausea and vomiting that were managed by a general practitioner. In spite of receiving antiemetics, her vomiting became intolerable which led to confusion and weakness.

**Figure 1:**
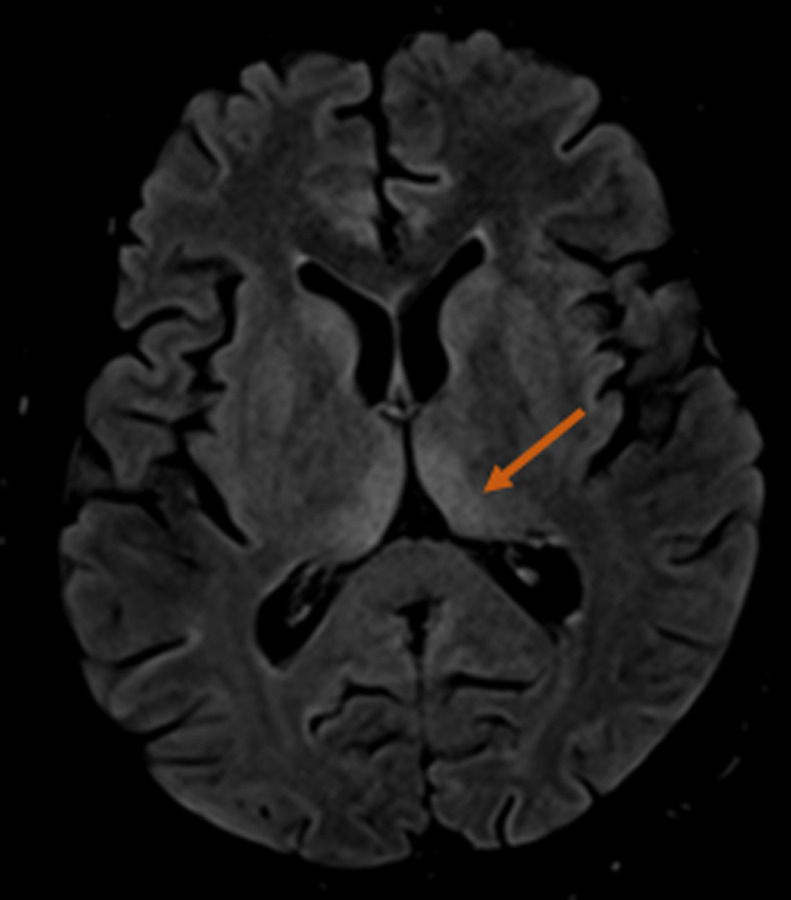
bilateral and symmetrical flair hyperintensity on the dorsomedial thalami

**Figure 2:**
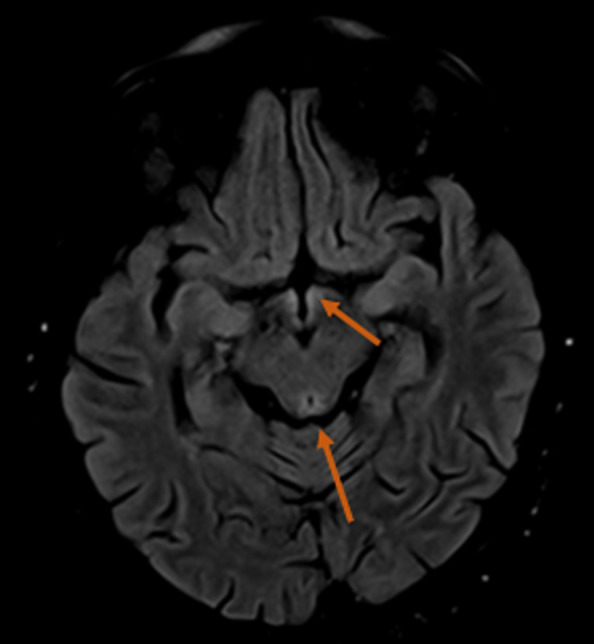
flair hyperintensity of the periaqueductal grey matter involving the tectal plate

**Figure 3:**
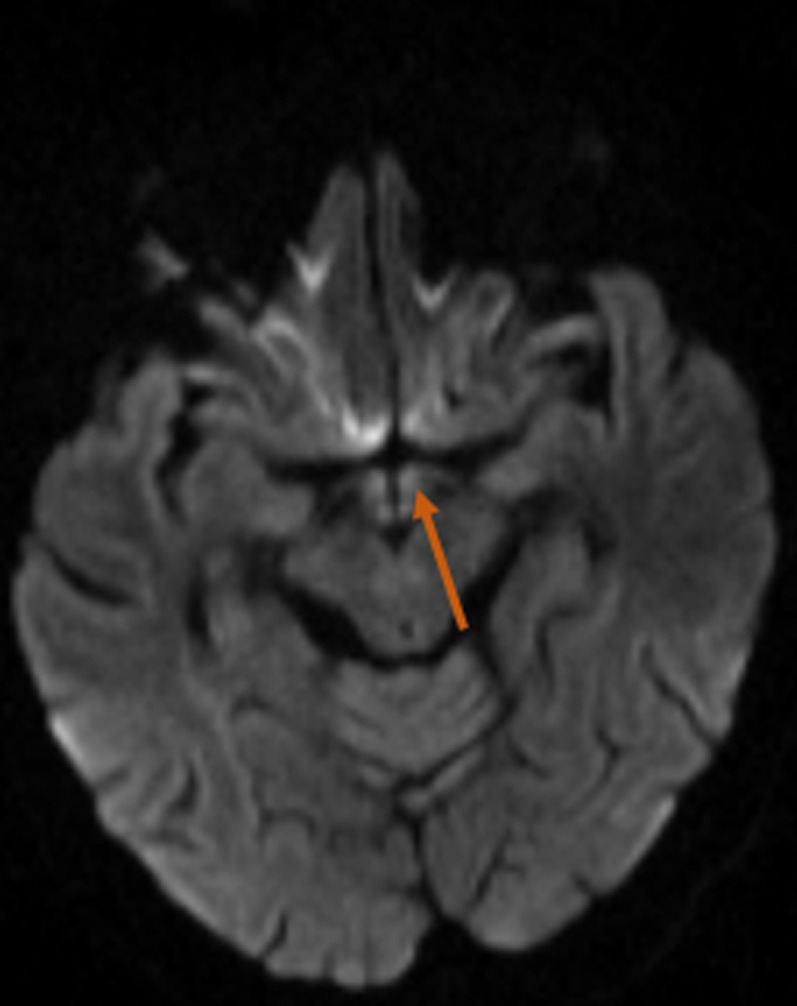
diffusion increased signal intensity of the mamillary bodies

**Diagnostic assessment:** ophthalmoscopy revealed bilateral disc swelling and superficial retinal hemorrhage. Laboratory tests showed the following anomalies: low hemoglobin and potassium levels (9.9 g/dl and 2.4mmol/l respectively) and elevated liver enzymes ASAT=102 ALAT=109. Ultrasound showed a single live intrauterine fetus. Brain MRI showed a diffuse T2/FLAIR hyperintensity in the thalamus (posteromedial zone, bilateral and symmetric) (Figure 1), the periaqueductal gray (Figure 2) and the mammillary bodies (Figure 3).

**Diagnosis:** these findings were highly evocative of Wernicke encephalopathy.

**Therapeutic interventions:** thiamine intravenous administration was initiated with a dose of 200mg/8 hours for 5 days, and then maintained with a dose of 100mg/12 hours. The patient also received intravenous glucose, saline solution with added potassium and antiemetic therapy.

**Follow-up and outcome of interventions:** the patient´s clinical condition began to improve and she no longer had any major deficits in memory or gait. On discharge, the patient was able to walk with assistance.

**Patient perspective:** our patient and her family were satisfied with our management and therapeutic protocol especially that the patient´s evolution was spectacular after thiamine administration.

**Informed consent:** the patient was informed of the specifics of her condition, of the reasons that made her case special and of the authors' interest in publishing it. She has given her informed consent to allow the authors to use her case for this case report.

## Discussion

Wernicke encephalopathy is a serious neuropsychiatric syndrome that was first described by Carl Wernicke in 1881 as polioencephalitis haemorrhagica superior with a resulting from thiamine deficiency [2]. Although alcoholism is the most common predisposing factor for this deficiency, WE can also occur in patients with nutritional deficiency states such as hyperemesis gravidarum, intestinal obstruction, bariatric surgery, chemotherapy, hemodialysis, and malignant diseases [3,4]. Thiamine is a water-soluble vitamin also known as vitamin B1. Its biologically active form, thiamine pyrophosphate (TPP), is a cofactor in macronutrient metabolism. In addition to its coenzyme roles, TPP plays a role in nerve structure and function as well as brain metabolism [3]. The body has approximately 18 days´ worth of thiamine stores. Therefore, signs of deficiency quickly develop with poor intake and/or absorption.

The classic clinical triad of WE syndrome consists of mental status changes, ophthalmoplegia, and gait ataxia [5]; however, this complete triad can be seen in just one-third of patients [5]. The most constant clinical finding is represented by mental status changes. These changes include confusional state, spatial disorientation, dizziness, drowsiness, apathy, cognitive impairment with disturbance in memory and inability to concentrate, coma, and even death. Such symptoms derive from an involvement of thalamic nuclei or mammillary bodies [5-7]. Among ocular disorders, complete ophthalmoplegia occurs rarely, while the most common ocular abnormality is nystagmus, usually horizontal. Other ophthalmic alterations include bilateral decreased visual acuity, diplopia, palsy of both lateral recti or other ocular muscles and conjugate-gaze palsies, retinal hemorrhage, papilledema, anisocoria, and ptosis [5-7]. Equilibrium disorders include gait ataxia that can vary from mild gait disturbance to a complete inability to stand. It results from an involvement of cerebellar vermis and vestibular dysfunction. Some patients also experience polyneuropathy and dysarthria [5,7].

This clinical presumption can be supported by brain MRI which is currently considered the most valuable method to confirm the diagnosis of Wernicke encephalopathy. Magnetic resonance imaging (MRI) has a sensitivity of only 53%, but its high specificity of 93% means that it can be used to rule out the disorder [5]. Magnetic resonance imaging (MRI) findings include bilateral, symmetric increased T2 signals in the paraventricular regions of the thalamus, hypothalamus, mammillary bodies, periaqueductal region, the floor of the fourth ventricle, and the cerebellum [4].

Wernicke encephalopathy is a medical emergency and any therapeutic delay may result in permanent neurological damage or death. The treatment of suspected or manifest WE is based on the administration of thiamine [4]. According to the European Federation of Neurological Societies and the Royal College of Physicians, 500 mg of parenteral thiamine should be given 3 times daily until acute symptoms of WE resolve [7]. Complete recovery isn't very common, and is only reported in cases where early recognition and thiamine supplementation were instituted. The fact that our patient presented the whole triad of WE with MRI signs then had a full recovery makes our case interesting and quite unique.

## Conclusion

Wernicke encephalopathy is one of the rare but potentially life-threatening neurological complications during pregnancy caused by hyperemesis gravidarum. This syndrome can lead to permanent brain damage and even death if not diagnosed and treated promptly. A full recovery isn't very common, but in our case, our patient had a spectacular evolution with no symptoms at her one-month follow-up. We should also keep in mind that WE can be prevented if a pregnant woman who suffers from severe and persistent vomiting is given thiamine supplementation without delay.
